# Provider comments reveal barriers to EHR nudge effectiveness: process evaluation of a null deprescribing trial

**DOI:** 10.1186/s43058-026-00983-2

**Published:** 2026-06-06

**Authors:** Ratnalekha V. N. Viswanadham, Hayley M. Belli, Tiffany Rose Martinez, Christina Wong, Saul B. Blecker, Andrea B. Troxel, Devin M. Mann

**Affiliations:** 1 Department of Population Health, NYU Grossman School of Medicine, 227 E 30th St, Sixth Floor, New York, NY 10016 USA; 2 MCIT, NYU Langone Health, New York, USA; 3 Department of Medicine, NYU Grossman School of Medicine, New York, USA

**Keywords:** Electronic health records, Behavioral economics, Implementation science, Diabetes, Nudge, Choosing wisely, Clinical decision support, Deimplementation

## Abstract

**Background:**

De-implementation—reducing low-value or harmful care—is critical but difficult in clinical practice. Clinical decision support (CDS) “nudges” in electronic health records (EHRs) aim to promote guideline-concordant deprescribing, but effects are inconsistent. In a pragmatic randomized controlled trial across a large health system, we tested a suite of EHR-based CDS nudges to support Choosing Wisely-aligned deprescribing of glycemic medications in older adults with type 2 diabetes. Although a prior pilot showed modest improvement in guideline concordance (5.1%), the full trial found no significant changes in prescribing; this process evaluation examines clinicians’ comments on alerts to explain why.

**Methods:**

We conducted a mixed-methods process evaluation of comments within EHR-based alerts from a null-result RCT that promoted Choosing Wisely deprescribing for older adults with type 2 diabetes. Among 66,634 alerts firing across EHR encounters (December 2016-July 2023), providers commented on 764 (1.2%). Two researchers independently coded comments using reflexive thematic analysis, identifying four themes (three negative). Exploratory logistic and multinomial regressions examined predictors of commenting, valence, and themes among acknowledged firings, adjusting for patient, provider, and encounter factors.

**Results:**

Thematic analysis of comments revealed three barriers to deprescribing: (1) disagreement with Choosing Wisely guidelines (308 comments, e.g., perceived low overtreatment risk); (2) workflow misalignment (203 comments, e.g., wrong provider responsibility); and (3) patient preferences (69 comments). Logistic regression showed multiple concurrent OPAs reduced action odds by 31.6% (OR 0.684, 95% CI 0.560–0.835); comments were 2.57 times more likely to be negative than positive (OR 2.565, 95% CI 1.637–4.018). Disparities in engagement were found, with female providers, patients, and socially vulnerable individuals less likely to comment.

**Conclusion:**

This process evaluation demonstrates scalable real-time feedback for clinical decision support refinement in de-implementation, with regressions identifying context-specific predictors. Provider disagreement, alert firings misaligned to workflows, and patient resistance hinder effectiveness. Future work should refine clinical decision support design to address complexity, enhance guideline explainability to build provider concordance, align with provider roles and workflows, and include patient-centered approaches.

**Trial registration:**

The NYU School of Medicine Institutional Review Board (i17-01308) approved the trial, which has the clinicaltrials.gov ID NCT04181307 (https://clinicaltrials.gov/study/NCT04181307), with a first record date of November 26, 2019.

**Supplementary Information:**

The online version contains supplementary material available at 10.1186/s43058-026-00983-2.

## Contributions to the literature

• We explore why EHR alerts for deprescribing failed in our diabetes trial through comments.• Comments were mostly negative, suggesting spotty engagement.• Providers disagreed with suggested guidelines, alerts were triggered at inappropriate times in the workflow, and patients resisted suggested changes.• Some providers engaged in alerts less for some patients (e.g., women, vulnerable groups), hinting at equity-related factors associated with EHR interactions.

## Introduction

Low-value care, including the overuse of tests and treatments that provide little or no benefit, is common and associated with preventable harm and unnecessary costs in healthcare systems [1]. One important manifestation of low-value care is potentially inappropriate glycemic management in older adults with type 2 diabetes, where intensive treatment can increase the risk of hypoglycemia, falls, and other adverse outcomes. Clinical guidelines now recommend less aggressive glycemic targets and deprescribing of certain medications for many older adults, particularly those with multimorbidity, frailty, or limited life expectancy [2, 3].

Deprescribing can be understood as a specific form of de-implementation, namely the planned and supervised process of reducing or discontinuing medications that may be unnecessary or harmful [4, 5]. Although the deimplementation of low-value practices has been the focus of an increasing number of trials and conceptual frameworks, substantial gaps remain in understanding how to support clinicians in changing long-standing prescribing behaviors in routine practice [4, 5]. Unlike implementation, which often focuses on building motivation and systems to support adoption of new practices, de-implementation must overcome deeply embedded habits, institutional norms, and fears of unintended consequences. For clinicians with competing demands and limited time, stopping an established practice is often more difficult than adopting a new one. Prescribing decisions are influenced by habits, clinical uncertainty, time pressure, organizational norms, and perceived expectations from patients and colleagues, all of which can lead to low-value care even when clinicians are aware of relevant guidelines [6, 7].

Traditional approaches to changing prescribing behavior, such as guideline dissemination and educational campaigns, have improved awareness but have not consistently produced practice change. The American Board of Internal Medicine launched the Choosing Wisely (CW) campaign to reduce overuse of tests and treatments and improve healthcare quality [1, 8]. While CW has raised awareness and promoted patient-provider conversations, widespread dissemination has not consistently reduced low-value care, highlighting the limitations of passive strategies. In response, implementation science and behavioral economics have turned to choice architecture and “nudges” to address factors beyond knowledge, such as default options, social norms, and cognitive load [9]. In healthcare, nudges delivered through clinical decision support (CDS) tools in electronic health records (EHRs) aim to provide timely prompts, suggested alternatives, or structured choices at the point of care, and have been used to promote appropriate prescribing and deprescribing [10]. CDS tools, for example, can flag inappropriate medication orders based on patient characteristics or prompt tailored recommendations during chart review, order entry, or patient messaging workflows [11, 12]. However, evidence suggests that nudges can sometimes fail to change behavior or have smaller-than-expected effects, underscoring the importance of understanding the mechanisms and contextual moderators of their impact [13–15]. Contextual factors such as alert timing, competing priorities, and trust in CDS shape the effectiveness of nudges [10, 16].

The present study is situated within a pragmatic randomized controlled trial (1:1 intervention versus usual care) that tested EHR-based nudges to reduce guideline-discordant glycemic treatment in older adults with type 2 diabetes [17]. In the trial, a suite of CDS-based behavioral interventions – collectively referred to as “Our Practice Advisories” (OPAs) and related tools – was designed using user-centered design methods and behavioral economics principles to encourage Choosing Wisely-aligned deprescribing behavior [18, 19]. The primary outcome, patient-level Choosing Wisely compliance, showed no statistically significant differences between intervention and control arms at 6 months (*p* = 0.638), 12 months (*p* = 0.620), or 18 months (*p* = 0.877). Despite a prior pilot study suggesting preliminary promise in guideline concordance, the full trial did not find a statistically significant effect of these nudges on prescribing outcomes [20]. This discrepancy between pilot and trial results underscores the need for process-oriented analyses to understand how clinicians engaged with the intervention and why behavior change did not occur at scale [21].

Process evaluations of complex interventions, as outlined in Medical Research Council guidelines, examine implementation, mechanisms, and context to interpret trial outcomes and inform future refinement of the intervention [22]. In this study, we conducted a process evaluation focusing on providers in the intervention arms who were exposed to EHR-based nudges. We analyzed clinician characteristics and free-text comments recorded in the EHR during interactions with alerts to examine how providers engaged with the intervention and how they justified their decisions to act—or not act—on deprescribing prompts [23, 24]. Prior work has shown that routinely collected EHR comment fields, entered during routine use rather than in dedicated usability testing, can reveal clinicians’ frustrations, workarounds, and perceptions of CDS logic, making them a useful source of implementation and design-relevant insight. Our aim was not to generalize to all nudges or clinical settings, but to generate context-specific insights into this particular intervention and trial.

Accordingly, the specific research question addressed in this study is: What do clinicians’ comments and interaction patterns with EHR-based deprescribing nudges reveal about why these nudges did not change prescribing behavior for older adults with type 2 diabetes (T2D)? By aligning with process evaluation principles, this analysis seeks to clarify how the intervention was received in practice, identify contextual and behavioral explanations for the null effect, and inform future design and implementation of EHR-based de-implementation strategies [25, 26].

## Methods

### Study aim, design, and setting

This study is a process evaluation of a null-result RCT (NCT04181307) that analyzes clinician comments on Our Practice Advisories (OPAs) to explain the limited intervention fidelity and reach. This study employed a mixed-methods approach to retrospectively evaluate and interpret clinicians’ comments, identifying the reasons clinicians agreed or disagreed with proposed care guidelines for de-prescribing T2D medications in older adult patients. A mixed-methods approach was necessary to capture both qualitative insights from provider comments regarding disagreements with the CDS nudge intervention and quantitative insights into the associations between the CDS and sentiments. This approach enables us to understand not only the effectiveness of the alerts but also the contextual factors that influence provider decisions and barriers to implementation. This study adheres to the GRAMMS checklist for mixed methods research, ensuring rigorous reporting of both the thematic analysis of provider comments and the quantitative analysis of alert firings [27, 28]. Analyses of OPA commenting and themes were conducted post hoc as exploratory secondary analyses and were not prespecified in the randomized trial protocol or statistical analysis plan. The study was conducted within the New York University Langone Health (NYULH) EHR and Epic system.

### RCT context

Details about the randomized controlled trial have been published previously in the trial protocol [17]. In summary, the intervention comprised six EHR-integrated clinical decision support tools, explicitly designed using principles of behavioral economics and developed through a user-centered design process. These tools included components such as a medication preference list that leveraged availability bias by placing guideline-concordant options (for example, metformin) at the top of the list, as well as non-interruptive navigator elements that increased the salience of Choosing Wisely recommendations while minimizing workflow disruption. These components align with published nudge taxonomies in healthcare (e.g., rearranging choice architecture via defaults and ordering, providing salient prompts at decision points, and emotional appeal) [29]. The CDS tools were refined through semi-structured interviews with key informants (*n* = 8), a multidisciplinary design-thinking workshop, and group interviews with clinicians at two vanguard clinics. An initial pilot at these clinics demonstrated an approximately 5.1%-point improvement in adherence to the Choosing Wisely guideline for older adults with diabetes.

These CDSs served as nudges, guiding provider decision-making without restricting providers’ autonomy in patient care (i.e., providers could override the CDS’s suggestions). The pilot implementation occurred in two vanguard clinics, while the RCT scaled deployment to the full NYULH primary care system (from 2 to 5 clinics approximately, expanding further); core CDS logic and components remained unchanged between pilot and RCT, though supplementary elements such as monthly peer comparison emails (leveraging social norms) were added during the RCT, with overall deployment spanning March 9, 2020, to September 8, 2021 (amid the acute phase of the COVID-19 pandemic).

**Fig. 1:**
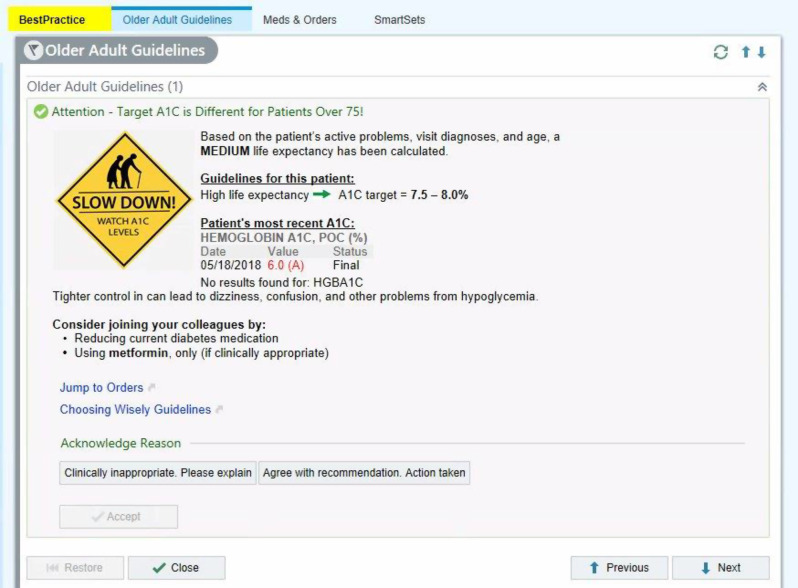
An example of the nudge of interest (formerly called a Best Practice Advisory and now Our Practice Advisory) to guide providers to prescribe medication appropriately

One type of clinical decision support implemented in the RCT was a built-in reminder in the Epic EHR system known as an “Our Practice Advisory” (OPA; formerly Best Practice Advisory and the nudge of interest in this study). OPAs are considered salience nudges because they make specific information (Choosing Wisely guidelines), options (deprescribing), or consequences (hypoglycemia) stand out to the intended user (clinicians), thereby directing their attention and influencing decisions (e.g., discussing medication management with the patient). OPAs triggered automatically during medication order entry and refill workflows for patients with an active type 2 diabetes diagnosis, who were at least 75 years old, had non-guideline-concordant glycemic regimens (e.g., sulfonylurea or insulin without metformin for low life expectancy), and no recent severe hypoglycemia. Nine unique OPAs combined specific recommendations – switching to metformin or lowering the non-metformin or metformin dose – with patient life expectancy categories (high: >10 years; medium: 3 - 10 years, low ≤ 3 years), computed via an EHR algorithm (age, comorbidities, and healthcare utilization history) and corresponding A1c targets (7–7.5%, 7.5–8%, 8–9%) [19, 30]. An example of such an OPA is shown in Fig. 1.

The EHR data recorded three dispositions for the alerts: ignored (dismissed without acknowledgement); acknowledged and contraindicated (opened without prescribing change); and acknowledged and completed (acknowledged with guideline-concordant deprescribing). Acknowledging providers in the intervention arm could optionally add free-text comments. The EHR records actions taken through the OPA as any steps the clinician takes beyond just clicking “acknowledge” or “dismiss.” Specifically, this included any EHR response code other than the six acknowledgment codes: “Acknowledge/Override Warning,” “No Action Taken,” “Accept Advisory [No Action Taken],” “Cancel Advisory,” “Deferred,” or “Deferred Advisory No Longer Applies.” All other responses (e.g., discontinuing an order, sending a message, or modifying a prescription) counted as action taken.

### Study population

The analytic sample included all physicians and advanced practice providers at NYULH who received one of the nine target OPAs related to glycemic medication titration for Type 2 diabetes (T2D) patients during encounters from December 22, 2016, to July 28, 2023; focused on intervention arm providers during RCT when applicable. The analytic dataset included all encounters in which at least one eligible OPA fired, regardless of whether a glycemic medication was prescribed, modified, or discontinued during that encounter. Thus, our unit of analysis is the alert firing (and associated encounter and clinician), rather than prescribing events themselves.

### Data source

Data were extracted from the NYULH EHR data warehouse via an SQL query for all firings between December 22, 2016, and July 28, 2023. For descriptive and analytic purposes, we distinguished two phases: the randomized trial intervention period (March 9, 2020 - September 8, 2021) and an outside-the-RCT period (before March 9, 2020, and after September 9, 2021, until July 2023).

Although the RCT formally tested a broader package of six CDS nudges, the specific OPA logic and alert content relevant to this study remained unchanged across the pilot, trial, and post-trial periods. We therefore pooled data from all three phases in the primary analyses for four reasons. First, the OPA algorithms (trigger criteria, message text, and recommended actions) underwent no substantive modification over time, so each alert firing represented the same underlying intervention. Second, the distribution of comment themes was largely stable across phases; multinomial regression comparing RCT-period comments with comments from the full observation period showed no statistically significant differences in the relative frequencies of the main themes, with the exception of a higher likelihood of workflow-related comments during the RCT period, consistent with increased clinical workload during the acute COVID-19 pandemic (Supplemental Table 2 and Supplemental Figure 1) [31]. Third, restricting analyses to the trial window alone would have yielded limited qualitative depth and sparse quantitative models.

### Variable selection

The primary data of interest were the free-text acknowledgment comments that providers could optionally enter when engaging with an alert (i.e., after opening and acknowledging the alert). Other data of interest for understanding the interaction with the alert included whether the provider engaged meaningfully with it and whether the provider left a comment on it.

Covariates were selected a priori based on prior clinical decision support literature examining predictors of alert engagement and response patterns, including patient demographics (for potential equity considerations), provider characteristics (for role and workload effects), and encounter features (for workflow and contextual factors) [32, 33] Patient-level covariates included age (continuous), self-identified race and ethnicity [34]. (categorized per U.S. Census Bureau definitions), sex (male, female, unspecified), and body mass index (BMI; continuous). Provider-level covariates included self-identified gender (male, female, unspecified), provider type (e.g., attending physician, nurse practitioner, physician assistant), and specialty (e.g., primary care, specialty internal medicine, surgery, and other). Encounter-level covariates included the number of other (non-study) nudge firings during the same patient-provider encounter (proxy for provider busyness and alert fatigue; rationale: higher co-firing volumes are associated with cognitive overload and lower response rates in CDS studies), the number of unique prior encounters in the patient-provider dyad (to account for relationship and clustering), and encounter type (e.g., ambulatory visit, telemedicine) [35, 36]. All models were adjusted for these factors to explore potential modifiers of commenting behavior and theme prevalence.

### Thematic coding

Two researchers (RVNV, HMB) independently reviewed and inductively coded all comments to identify initial patterns and general takeaways (e.g., primary reason for agreement/disagreement). RVNV completed coding first, after which the codes were concealed; HMB then coded independently. Coders then compared results collaboratively, achieving high agreement on four overarching themes, with three negative themes each containing distinct subthemes (e.g., guideline-disagreement subthemes). Discrepancies were resolved through discussion until consensus was reached; multiple codes per comment were permitted. Themes were classified as positive (agreement or action aligned with OPA) or negative (disagreement or no action). This followed principles of reflexive thematic analysis [37].

### Statistical analyses

All quantitative analyses were conducted post hoc to complement the primary thematic findings and to support a process evaluation of how clinicians engaged with alerts; they were not prespecified in the RCT protocol or statistical analysis plan. Analyses were performed using RStudio (version 4.3.2). No formal power calculations were performed because the analyses were exploratory and hypothesis-generating, based on sparse comment data (approximately 1.2% of 67,412 firings). Our goal was to describe contextual patterns and potential selection mechanisms, not to make definitive causal or confirmatory inferences.

We summarized patient, provider, encounter, and OPA characteristics overall and by commenting status and action type. Comment counts and proportions were tabulated by theme and subtheme. To assess whether themes identified from comments during the RCT were generalizable to the broader period of alert use, we conducted a multinomial logistic regression restricted to commented alerts, comparing the distribution of themes during the RCT versus the full alert-firing period (reference: positive comments). Model fit was assessed using the change in the Bayesian Information Criterion (${\rm{\Delta }}$BIC) relative to intercept-only null models, with differences > 10 indicating strong evidence of improvement, 6–10 strong evidence, 2–6 positive evidence, and 0–2 negligible differences [38].

We used three regressions with encounter-level covariates to quantify how contextual features of encounters were associated with alert engagement and comment valence:

1. Action on alert: A logistic regression with outcome “action taken on alert recommendation” estimated associations between encounter context (e.g., number of other alerts fired during the encounter as a proxy for alert burden, visit type, day of the week, alert type) and the likelihood that clinicians acted on the nudge. The EHR classified clinicians as having “acted on the alert” if they took any step beyond just dismissing or acknowledging it (e.g., discontinuing a medication, sending a follow-up message, or modifying an order). Simple dismissals or overrides without further action were classified as no action. This addressed how real-world workflow and alert load may be associated with lower nudge effectiveness.
2. Commenting behavior (selection into comments): A second logistic regression with outcome “comment left versus not” examined whether the same encounter-level factors were associated with clinicians choosing to leave a comment. This regression was motivated by the need to characterize selection into the comment sample and to inform how representative the qualitative data are of all alert firings.
3. Negative versus positive comments (engagement valence): Among encounters with a comment, a third logistic regression with outcome “negative versus positive comment” assessed whether encounter context was associated with expressing disagreement or frustration rather than agreement. This analysis associated workflow context to the valence of feedback about the nudges.

For the first two encounter-level models, we accounted for clustering at both the provider and patient level to reflect that the individual patients could contribute multiple encounters and that individual providers could see multiple patients, leading to non-independent observations. We used cluster-robust (sandwich) standard errors with two-way clustering by provider and patient, and calculated intraclass correlation coefficients (ICCs) at the provider and patient levels. Results are reported as odds ratios (ORs) with 95% Wald-type confidence intervals derived via the delta method from the clustered standard errors. For each logistic model, we computed McFadden’s pseudo-R^2^ as a descriptive measure of model fit.

To examine whether reasons for rejecting the alert varied by clinical scenario, we fit a multinomial logistic regression model on commented alerts, with the outcome variable the main comment theme (rejection themes versus acceptance or a positive comment), and predictors including alert recommendation type and patient life expectancy category. This analysis tested the hypothesis that particular rejection themes (e.g., workflow concerns versus guideline disagreement) would be common for specific combinations of recommendation content and prognosis.

Because comments were sparse and potentially non-representative, we conducted additional regressions to assess which providers and patients were more or less likely to appear in the comment data. Logistic regressions evaluated whether leaving any comment (versus none) and whether leaving a negative (versus positive) comment among commenters were associated with provider characteristics (e.g., gender, specialty, and clinician type). Parallel logistic regressions evaluated whether having any comment (versus none) and having a negative (versus positive) comment were associated with patient characteristics (e.g., sex, race/ethnicity, social vulnerability, and frequency of encounters). No clustering was performed for patient- or provider-level regressions to avoid perfect prediction or separation issues arising from the one-to-one structure of the data at those levels (i.e., each provider/patient observation is unique within the comment sample).

## Results

### Descriptive statistics

During the full implementation period (December 22, 2016–July 28, 2023), the glycemic OPA fired 66,634 times across 39,654 unique encounters involving 373 unique providers. Providers left a total of 764 free‑text comments on these alerts (1.15%). During the randomized trial (RCT) period (March 9, 2020–September 8, 2021), the OPA fired 24,399 times, and providers left 207 comments (0.85%). The alerts fired 10,457 times during the pilot phase (before the RCT) and 31,477 times after the RCT.

Across all OPA firings, clinicians acted on and through the OPA recommendation in 54 encounters (0.2%) and did not act in 39,645 encounters (99.8%). Comments were more commonly left on alerts where the recommendation was not followed: providers commented on 758 of 39,645 non‑acted alerts and on 1 of 5 acted alerts. For the thematic analysis, we focused on comments left during the full implementation period, regardless of the EHR recording action taken.

Patients exposed to the alert were older adults with type 2 diabetes, with a mean age of 81.4 years (SE 0.0423); 52.7% were female, and 69.7% were non‑Hispanic/Latino White (Table 1). Most alerts occurred in outpatient encounters; 71.7% were with general internal medicine providers. Providers leaving comments were similar in age, sex, and specialty distribution to the overall provider population, with exceptions noted in the representativeness analyses below. An expanded table of descriptive statistics is provided in Supplemental Table 1.

**Table 1:** Descriptive statistics of patients and providers based on comments left or not

	Any Alert Firings	Alert Firings Without Acknowledgment Comments	Alert Firings With Acknowledgment Comments
Patient Characteristics			
Unique Patients	7663	7610 (92.6%)	564 (7.4%)
Female	3986 (52.7%)	3965 (52.7%)	267 (47.7%)
Male	3583 (47.3%)	3552 (47.2%)	293 (52.3%)
Non-Hispanic/Latino White	5339 (69.7%)	5303 (69.7%)	381 (67.6%)
Non-Hispanic/Latino Black	879 (11.5%)	874 (11.5%)	70 (12.4%)
Asian	336 (4.38%)	336 (4.42%)	22 (3.9%)
Hispanic/Latino	308 (4.02%)	308 (4.05%)	29 (5.14%)
Multiple Races	31 (0.405%)	30 (0.394%)	4 (0.709%)
Native American/Native Alaskan/Native Hawaiian	15 (0.196%)	15 (0.197%)	0 (0%)
Other Race/Ethnicity	8 (0.104%)	8 (0.105%)	1 (0.177%)
Unknown	748 (9.76%)	737 (9.68%)	57 (10.1%)
Age – years (Mean (SE), Median [IQR])	81.4 (0.0423) 80.2 [77.2, 84.3]	81.4 (0.0424) 80.2 [77.2, 84.3]	81.8 (0.192) 80.8 [78, 84.6]
Number of Encounters per Patient (Mean[SE], Median [IQR])	5.35 (0.0497) 3 [2, 7]	5.38 (0.0498) 3 [2, 7]	7.05 (0.273) 5 [2, 9]
Provider Characteristics			
Unique Providers	373	372	82
Female	137 (36.7%)	137 (36.7%)	28 (34.1%)
Male	121	121	31
Other/Unknown	115	1149	23
Physician (MD or DO)	289 (77.3%)	288 (77.2%)	69 (84.1%)
Nurse Practitioner (NP)	65 (17.4%)	65 (17.4%)	10 (12.2%)
Physician Assistant (PA)	18 (4.81%)	18 (4.83%)	3 (3.66%)
Registered Nurse (RN)	1 (0.267%)	1 (0.268%)	0 (0%)
Internal Medicine - General	268 (71.7%)	268 (71.8%)	61 (74.4%)
Internal Medicine - Specialty	88 (23.5%)	87 (23.3%)	18 (22%)
Other Specialties	16 (4.28%)	16 (4.29%)	2 (2.44%)
Surgery	3 (0.802%)	3 (0.804%)	1 (1.22%)
Encounter and Alert Characteristics			
Unique Encounters	39654	39132	763
Alerts Fired Per Encounter (Mean [SE], Median [IQR])	3.05 (0.00834) 3 [2, 4]	3.07 (0.0084) 3 [2, 4]	2.3 (0.0538) 2 [1, 3]
Number of Alert Firings	66634	65570	764
Patient Life Expectancy: High	40329 (60.8%)	39821 (60.7%)	508 (66.5%)
Patient Life Expectancy: Medium	24441 (36.8%)	24204 (36.9%)	237 (31%)
Patient Life Expectancy: Low	1564 (2.36%)	1545 (2.36%)	19 (2.49%)
Alert Suggestion: Lower Metformin	35474 (53.5%)	35035 (53.4%)	439 (57.5%)
Alert Suggestion: Switch from Non-Metformin to Metformin	26669 (40.2%)	26395 (40.3%)	274 (35.9%)
Alert Suggestion: Reduced Non-Metformin	4191 (6.32%)	4140 (6.31%)	51 (6.68%)
No Action Taken Through Alert	66266	65507	759
Action Taken Through Alert	67	62	5

### Thematic analysis of provider comments

Of the 764 comments left on nudge firings, 759 were associated with encounters where clinicians acted on the recommendation, and 5 comments were associated with encounters where clinicians did not act. Comments on non‑acted recommendations were more frequent and typically provided reasons for rejecting the recommendation; comments on acted alerts that mostly documented agreement with the guideline described how the recommendation was implemented, like “Metformin stopped for now patient will continue Prandin in the interim and slowly taper off”, or combined with next steps before decisions like “meds reduced to twice daily await labs.” Given the small number and limited thematic variation of comments on acted alerts, the primary thematic analysis focused on comments associated with non‑acted recommendations. Three major themes characterized reasons for not acting on the OPA (Table 2). Comments frequently mapped to more than one subtheme.

**Table 2:** Main themes, subthemes, and representative comments during the entire OPA activation period

Theme	Provider disagreement with suggestion	Workflow misalignment	Patient resistance	Provider utilized OPA advice.
Number of Comments (N Comments = 565)	308 (54.5%)	203 (36.0%)	69 (12.2%)	208 (36.8%)
Sub-themes	No symptoms or doing well, so no reason to take action. (*N* = 160) Clinician doesn’t agree with nudge or guidelines. (*N* = 78) Other clinical reason to not take action. (*N* = 76) A1c is secondary objective (e.g., primary weight loss, etc.) (*N* = 9)	Followed by another clinician (e.g., PCP, endocrinologist) so not their responsibility (*N* = 125) Awaiting new lab result/will re-evaluate. (*N* = 89)	N/A	N/A
Representative Comments	“She has no hypoglycemia and FS have been normal or high”	“DM followed by PCP”	“patient refuses to reduce meds”	
	“patient with hyperglycemia with BS at home 200–400” “look at his glucose; these prompts are silly”			Metformin stopped for now patient will continue Prandin in the interim and slowly taper off
	doing well with low dose metformin which poses no risk	“await labs”	“Patient has been reluctant to stop his medication despite the guidelines which have been discussed and he clearly understands however since he is tolerating his regimen well with no hypoglycemic episodes he asks to continue treatment.”	dose lowered to once daily from twice daily.
	“multiple co-morbities with vascular disease and CKD which require better glycemic control”			meds reduced to twice daily await labs
	“CVD secondary prevention.”	“Will consider at next appointment”	“Patient wants to stay on medication optimize glycemic control”	
Number of unique providers providing this type of comment	59	41	27	47
Number of unique patients for this type of comment	260	150	60	189
OPA firings (N)				
High Life Expectancy OPAs	219	120	46	136
Medium Life Expectancy OPAs	80	82	30	65
Low Life Expectancy OPAs	9	1	3	7
OPA Suggestion: Lower Metformin	183	96	50	123
OPA Suggestion: Switch from Non-Metformin to Metformin	103	93	15	70
OPA Suggestion: Reduced Non-Metformin	22	13	4	15

The most frequent main theme was provider disagreement with the Choosing Wisely guideline or OPA recommendation (Theme 1; 308 comments), comprising three subthemes. The first subtheme was a perceived risk of overtreatment. In 160 comments, clinicians noted that the patient had no symptoms of hypoglycemia, no complications related to tight glycemic control, and was clinically stable. In these cases, providers described the current regimen as well-tolerated and did not perceive the patient as at risk of adverse effects from continued intensive glycemic control. The second subtheme was disagreement with the CW guidelines. In 78 comments, clinicians explicitly questioned the relevance or appropriateness of the OPA, independent of the specific patient. These comments included statements that the alert was “not helpful” or did not align with the clinician’s assessment of best practice. The third subtheme was that there were other clinical reasons not to follow the recommendation. In 76 comments, providers cited comorbidities or clinical histories as reasons not to follow the recommendation, such as chronic kidney disease, anemia, coronary artery disease, or prior poor response to the suggested regimen. The third subtheme presented was competing clinical priorities. In 9 comments, providers indicated that A1C control was a secondary objective relative to other clinical issues and thus chose not to change the medication regimen at that encounter.

The second major theme was workflow misalignment, with two subthemes (Theme 2; 203 comments). The first subtheme was that the recommendation was attributed to another provider. In 125 comments, clinicians stated that glycemic management was under the purview of another provider, often the patient’s primary care clinician or endocrinologist; therefore, they did not adjust the regimen in response to the alert. The second predominant subtheme was that the provider was waiting for additional information or a visit. In 89 comments, clinicians indicated that they were waiting for updated laboratory results or planned to discuss medication changes at a future visit before modifying treatment. In these instances, providers deferred action rather than immediately implementing the recommendation.

The third theme, patient resistance as a barrier to deprescribing (Theme 3; 69 comments), had no subthemes. In these comments, clinicians indicated that patients declined or resisted recommendations to reduce or stop medications, even when clinicians considered deprescribing appropriate. Examples included comments such as “patient refuses to reduce meds,” “patient has been reluctant to stop his medication despite the guidelines, which have been discussed,” and “patient wants to stay on medication to optimize glycemic control.” These comments described situations in which clinicians perceived the OPA recommendation as clinically acceptable but did not implement it due to patient choice.

### Associations between response to OPAs and encounter characteristics

Multinomial logistic regression was used to compare themes of negative comments during the RCT period with those from the full implementation period. Providers were more likely to indicate that the OPA was placed incorrectly in the workflow during the RCT period than during the broader implementation period. No other significant differences in theme distributions were detected. The multinomial logistic model showed a weak fit (${\rm{\Delta }}$BIC = 1.395). These findings are shown in Supplemental Figure 1 and Supplemental Table 2.

**Fig. 2:**
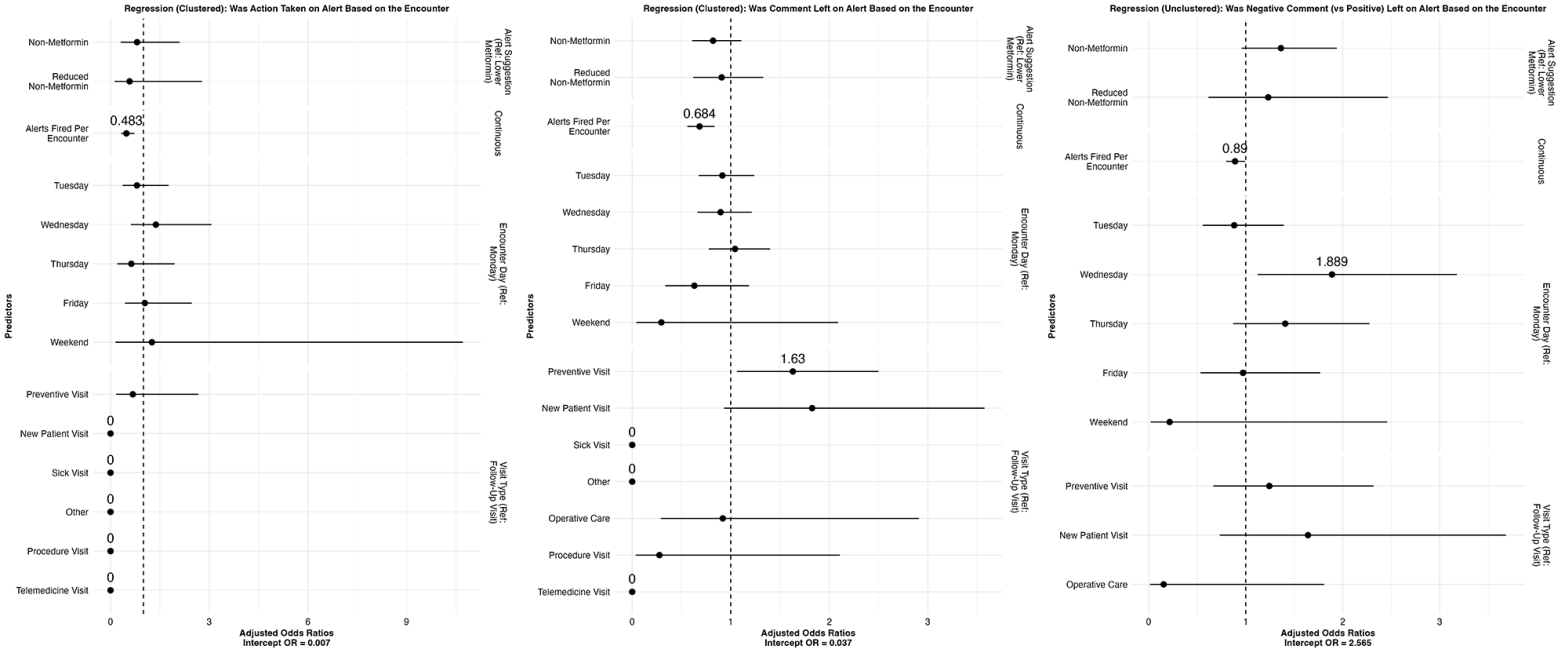
Odds ratios on associations between providers leaving comments on OPAs (left) or providers leaving negative comments (right) and encounter characteristics. Significant odds ratios have values labeled

Three logistic regressions examined associations between alert behavior and encounter‑level characteristics. Across encounters, the estimated probability of taking action on an alert recommendation was 0.69% (OR = 0.0069, 95% OR CI = 0.00289–0.0130). Each additional OPA fired within an encounter was associated with a 51.7% reduction in the odds of acting on the recommendation (OR = 0.469, 95% OR CI = 0.365–0.603). Odds ratios are displayed in Fig. 2a, with full model estimates in Supplemental Table 3.

Across encounters, the estimated probability of leaving a comment on an OPA recommendation was 3.7% (OR = 0.037, 95% OR CI = 0.015–0.089). Each additional alert fired within an encounter was associated with a 31.6% reduction in the odds of leaving a comment on the alert (OR = 0.684, 95% OR CI = 0.560–0.835). Comments were more likely to be left during preventive visits (OR = 1.630, 95% OR CI = 1.063–2.499) than during other visits, like follow-up or sick visits. Odds ratios are displayed in Fig. 2b, with full model estimates in Supplemental Table 4.

Among encounters with comments, clinicians were more likely to leave negative than positive comments (OR = 2.565; 95% OR CI = 1.637–4.018). Each additional OPA fired within an encounter was associated with a 11.0% decrease in the odds of leaving a negative comment (OR = 0.890, 95% OR CI = 0.798–0.993). Compared with Mondays, negative comments were 88.9% more likely on Wednesdays (OR = 1.889, 95% OR CI = 1.123–3.177). Odds ratios are displayed in Fig. 2 (part c, right), with full model estimates in Supplemental Table 5. Other encounter‑level characteristics, including most days of the week, alert suggestions, and encounter type, were not significantly associated with either acting on the recommendation, leaving a comment, or leaving a negative comment.

### Associations between rejection themes and clinical context

**Fig. 3:**
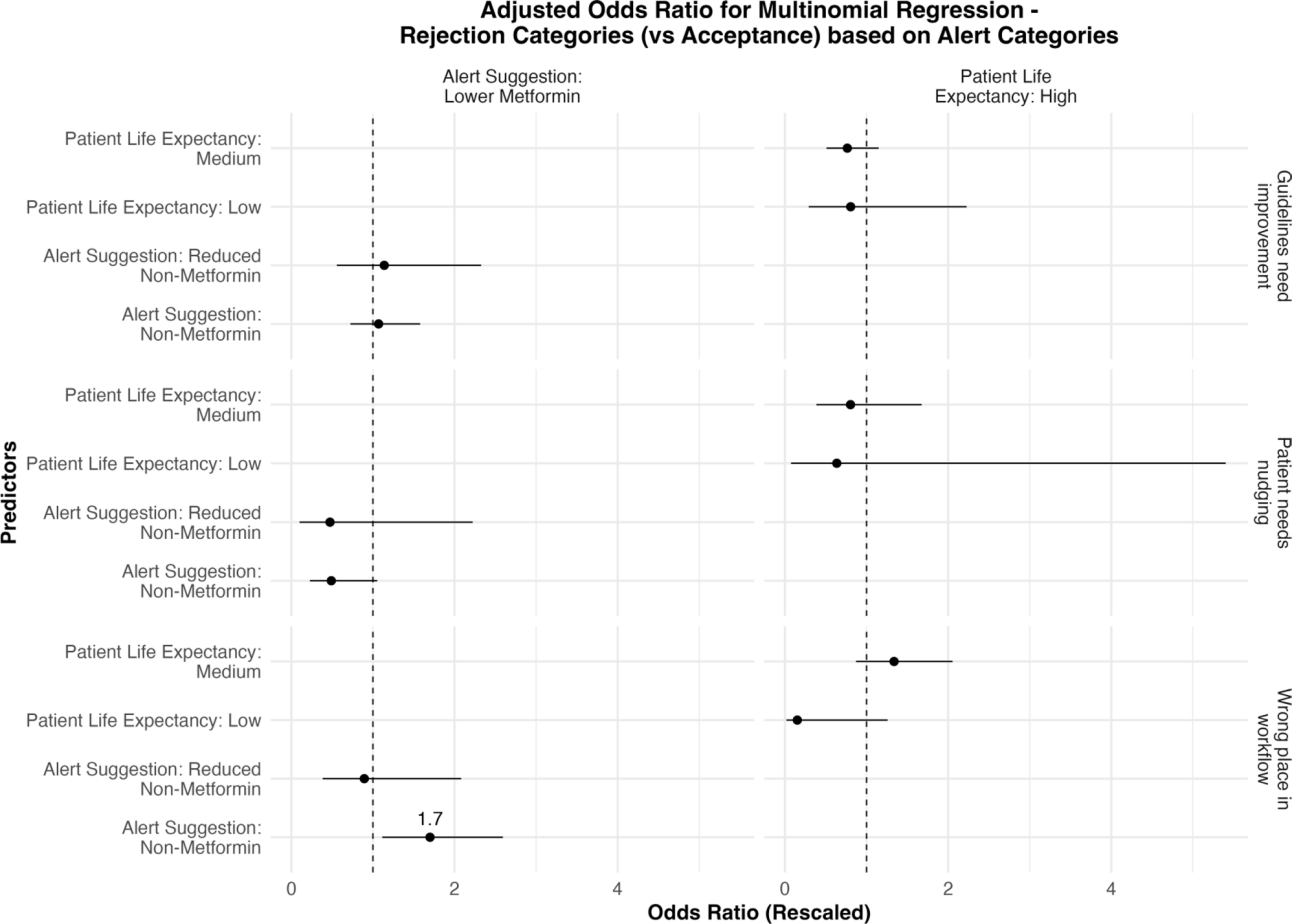
Multinomial logistic regression to evaluate the association between rejection and OPA themes

Multinomial logistic regression was used to assess whether rejection themes (versus agreement with the OPA recommendation) were associated with clinical context, including recommendation type and estimated patient life expectancy. Alert suggestion strength and clinician life expectancy expectations substantially improved the prediction of theme selection (${\rm{\Delta }}$BIC = −47.5 vs. intercept-only null), providing strong evidence of model fit beyond baseline proportions. Relative to comments indicating agreement with the OPA recommendation, comments stating that the guideline needed improvement were more likely (OR = 1.31, 95% OR CI = 1.13–1.53). In contrast, comments indicating that the OPA was placed in the wrong location in the workflow were less likely than agreement comments (OR = 0.374, 95% OR CI = 0.280–0.499). Comments about inappropriate workflow placement were less common when the patient’s estimated life expectancy was low (OR = 0.427; 95% OR CI = 0.265–0.688) than when it was high. Comments indicating that a patient needed encouragement to change medications were less common for patients with moderate vs. high estimated life expectancy (OR = 0.745, 95% OR CI = 0.604–0.918). Results are summarized in Fig. 3 and Supplemental Table 6.

### Representativeness analyses

**Fig. 4:**
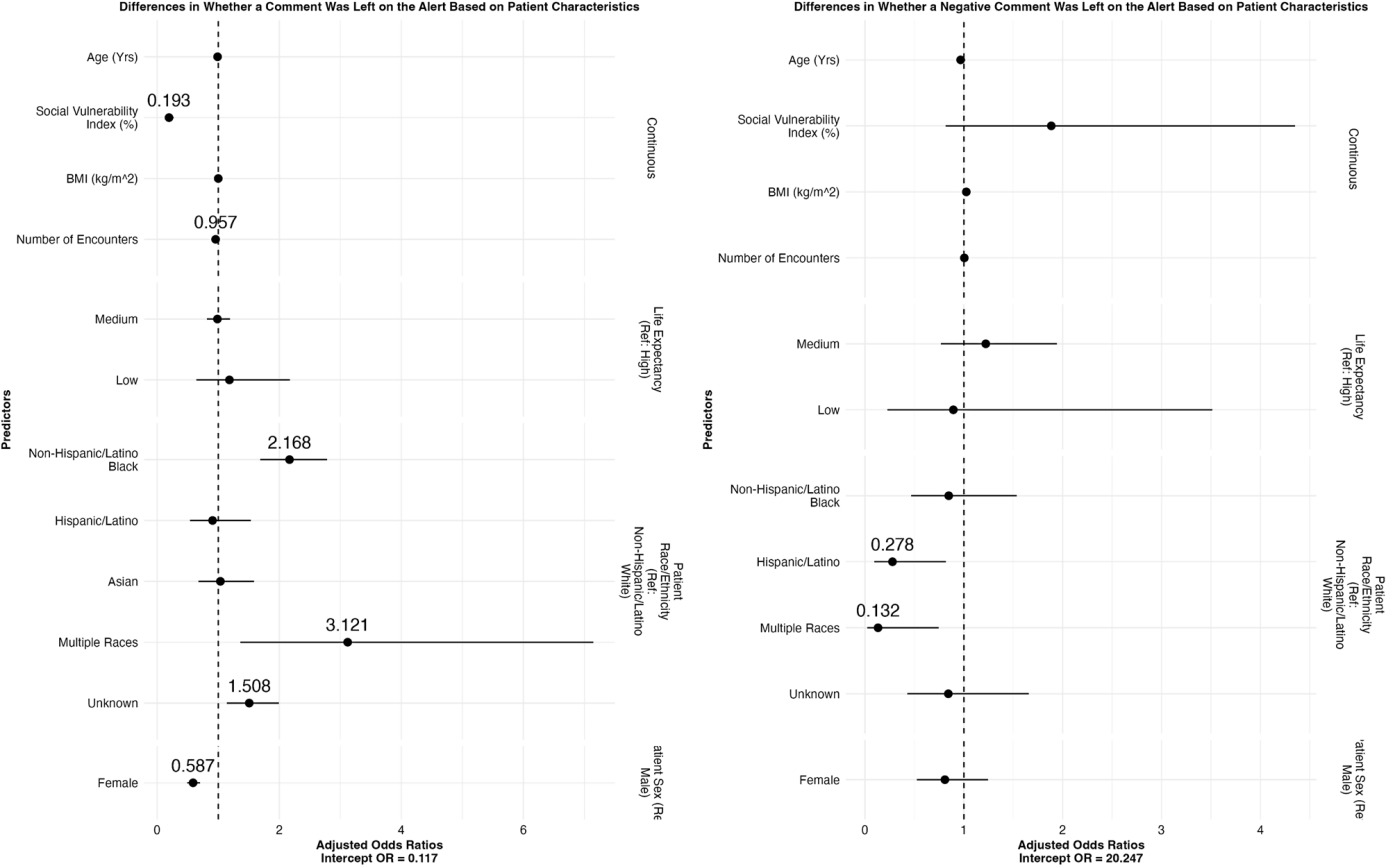
Odds ratios on associations between providers leaving comments on OPAs (left) or providers leaving negative comments (right) on patient characteristics

**Fig. 5:**
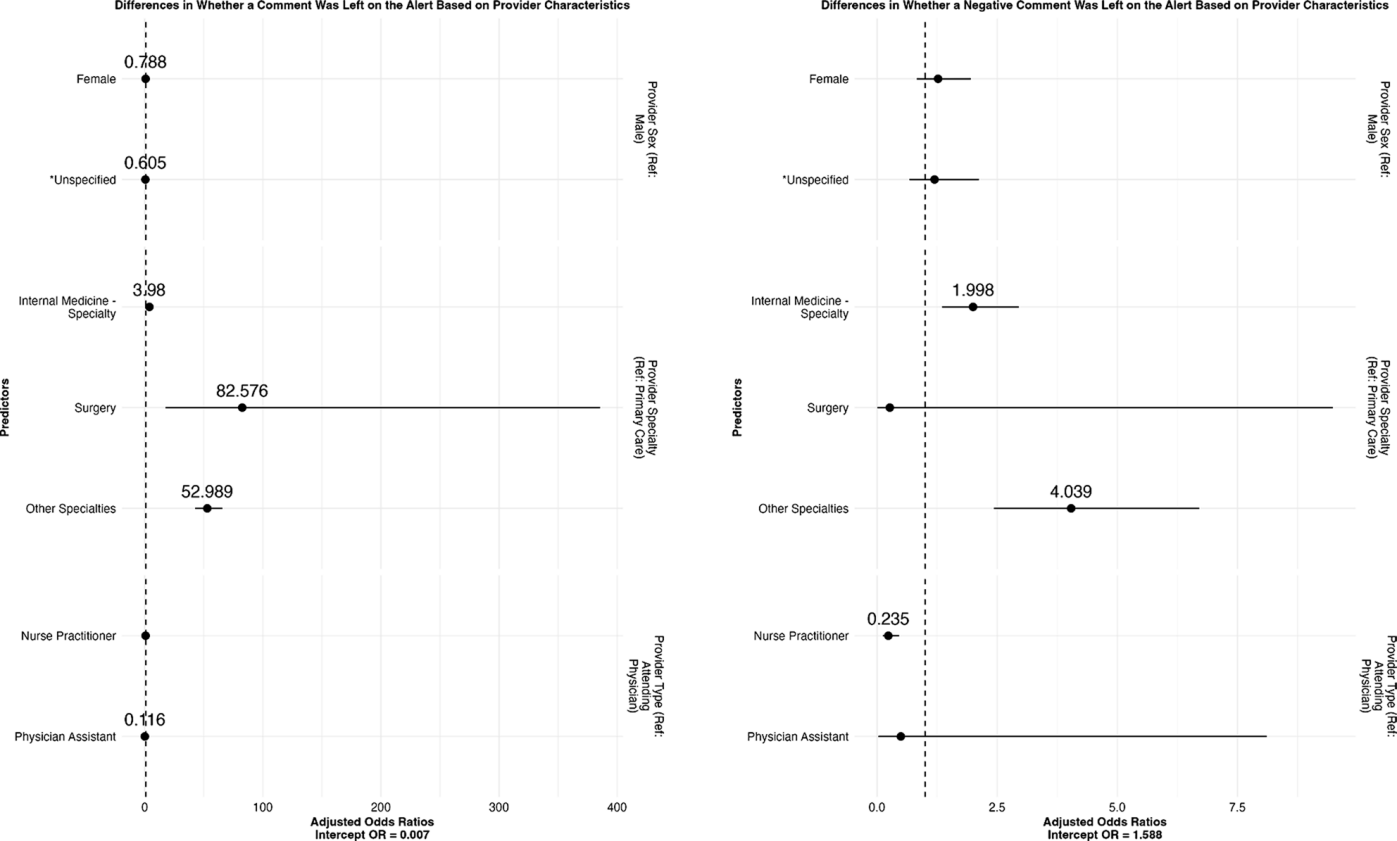
Odds ratios on associations between providers leaving comments on OPAs (left) or providers leaving negative comments (right) on provider characteristics

Logistic regressions were used to assess whether the populations of patients for whom comments were left and providers who commented on the OPAs were representative of those exposed to OPAs overall and whether comment valence varied by these characteristics. Results are presented in Fig. 4 (patients) and Fig. 5 (providers) and Supplemental Tables 7, 8, 9, and 10.

Among patients, female patients were less likely than male patients to have comments left on their OPA firings (OR 0.587, 95% OR CI = 0.492–0.701), but the odds of negative (vs. positive) comments did not differ significantly by patient sex (OR 0.808, 95% OR CI = 0.525–1.24). Several racial and ethnic minority groups, compared with non‑Hispanic White patients, were more likely to have comments associated with their OPA firings, although the odds of negative vs. positive comments did not differ significantly by race/ethnicity. Patients living in areas of higher social vulnerability were less likely to have comments associated with their OPAs (OR 0.193, 95% OR CI = 0.138–0.270). Patients with more prior encounters with the same provider had slightly lower odds of leaving a comment on an OPA (OR = 0.957 per additional encounter; 95% OR CI = 0.945–0.970).

Among providers, female clinicians were less likely than male clinicians to leave any comment on an OPA (OR = 0.788, 95% OR CI = 0.652–0.954). When comments were left, clinicians were more likely to leave negative than positive comments (OR = 1.59 for negative vs. positive, 95% OR CI = 1.15–2.19). Subspecialty providers were more likely than general internal medicine providers to leave comments overall and to leave negative comments.

## Discussion

We conducted a process evaluation using mixed quantitative and qualitative methods to identify potential barriers to the effectiveness of CDS in promoting deprescribing recommendations for glycemic medications in older adults with type 2 diabetes (T2D).

Clinicians had low likelihoods of acting upon alerts and leaving comments within alerts. Should they leave comments, they were more likely to leave negative than positive comments. In exploratory analyses, receiving more alerts during an encounter was significantly associated with interacting with alerts and providing comments and feedback. Clinicians’ free-text comments on alerts (salience nudges) in a large academic health system’s EHR revealed three key implementation barriers.

First, clinicians expressed disagreement with CW guidelines or OPA recommendations, citing perceived risks of undertreatment, inapplicability, comorbidities, or competing priorities. Providers perceived the CW recommendations as insufficiently specific for patients with multimorbidity or non–T2D medication indications, which may limit impact even when guidelines are disseminated and embedded in CDS, consistent with long‑standing evidence that guidelines alone often produce modest behavior change [39]. Their inaction also aligns with evidence that individuals are less likely to trust algorithmic recommendations when those recommendations conflict with their own decisions in similar contexts [40]. Comments highlighted guideline ambiguity for complex cases (e.g., non-diabetes indications and comorbidities), suggesting explainability could enhance applicability while preserving autonomy.

Second, nudges were placed in the wrong location within the prescribing workflow, either with the non-responsible provider or before the provider had the necessary information to act in accordance with the advisory. Engineering CDS to focus on the point-of-contact provider and alerting the provider at the appropriate decision-making point could increase CDS utilization rates. EHR metadata can provide insights into workflow targeting. Further research can explore how EHR metadata, which describes granular interactions with the EHR, can inform CDS design within clinical workflows to capture providers’ attention and support intended behavior change. Similarly, the CDS for this RCT had low utilization and action rates, as identified by the post-hoc analyses. CDS requires thoughtful design and integration with other healthcare implementations to ensure utilization and effectiveness, as alert fatigue and clinician desensitization to CDS can lead to overlooking critical alerts.

Clinicians noted patient refusal to deprescribe despite provider endorsement, often citing mortality salience in CW discussions or empowerment gaps. Comments highlighted guideline information that patients fundamentally disagreed with, such as risks of hypoglycemia or regimen changes. Potential strategies include independent patient nudges to initiate discussions, behavioral “boosts” that combine empowerment with nudges, or EHR-integrated shared decision-making tools tailored to lived experience [41, 42].

Associations between alert engagement and patient characteristics showed that comments were more likely to be left for patients who identified as non-Hispanic/Latino Black and less likely for patients who identified as female, were more socially vulnerable, and had more encounters with their provider. Female providers were less likely to comment on alerts than male providers, and specialty providers were more likely to comment than general internal medicine providers. Providers were more likely to leave negative comments, with specialty providers more likely to do so than general internal medicine providers. These identified associations can contribute to the growing literature of EHR documentation and usage differences by provider gender and specialty [43, 44].

## Strengths

This process evaluation leverages a large dataset to provide real-time clinician feedback via built-in comments, a scalable method informed by user-centered pilot design that enables iterative CDS refinement and exemplifies learning health system principles [22]. Our themes—guideline disagreement, workflow misalignment, and patient preferences—mirror determinants of de-implementation described in prior multi-method work, including clinician beliefs, contextual constraints, and patient factors [45]. Our results highlight practical challenges in operationalizing Choosing Wisely recommendations for glycemic control in older adults, echoing concerns about the difficulty of de‑implementing low‑value care within CW campaigns [46]. Despite low rates, 779 comments, along with regression analyses, yield predictors despite null effects, which contrasted pilot gains and highlighted equity gaps in CDS reach. We also use process evaluation to explore possible hypotheses regarding null results from RCTs, with the aim of improving future RCTs in fields such as de-implementation, behavioral economics-based implementations in EHRs, prescribing behavior, and EHR design.

## Limitations

The analyses have limitations. First, low alert acknowledgment (1.15% comments) introduces selection bias; the findings represent only commenting providers. Second, pooling pilot and RCT data assumes comparability despite the COVID-19 pandemic disruption; we pooled data because of sparsity and because the CDS design did not change between the pilot, RCT, and post-RCT data. Third, results from one academic center limit generalizability. Future research hopes that the methods, data, and identified rejection themes can contribute to the broader research scopes of CDS effectiveness, health services research, and implementation science.

## Conclusion

This process evaluation of a cluster-randomized trial demonstrates that clinician comments on EHR-based CDS alerts (OPAs) offer actionable insights into implementation barriers to deprescribing glycemic medications in older adults with type 2 diabetes. This process evaluation of a cluster-randomized trial demonstrates that clinician comments on EHR-based CDS alerts (OPAs) offer actionable insights into implementation barriers for deprescribing glycemic medications in older adults with type 2 diabetes. Three themes emerged from 764 comments across 66,634 OPAs: guideline disagreement and lack of clarity, workflow misalignment, and patient-related hesitancy. Logistic regressions identified encounter- and provider-level predictors of commenting and negativity, informing context-specific tailoring of CDS design, timing, and explainability. These findings contribute a scalable method for real-time feedback in CDS evaluation, advancing implementation science for de-implementation in EHR systems.

## Electronic supplementary material

Below is the link to the electronic supplementary material.

Supplementary Material 1

Supplementary Material 2

## Data Availability

Deidentified data may be available upon request.

## Abbreviations

A1C: Hemoglobin A1c
ABIM: American Board of Internal Medicine
${\rm{\Delta }}$BIC: Change in the Bayesian Information Criterion
CAD: coronary artery disease
CDS: Clinical Decision Support
CKD: Chronic Kidney Disease
CW: Choosing Wisely Guidelines
T2D: Diabetes mellitus type 2
EHR: Electronic health record
ICC: intraclass correlation coefficient
IQR: interquartile range
NYU: New York University
NYULH: New York University Langone Health (Medical Center)
OPA: Our Practice Advisory
OR: Odds ratio
RCT: randomized controlled trial
SE: standard error of the mean
ST: supplemental table
