# Supplementary material for: Provider comments reveal barriers to EHR nudge effectiveness: process evaluation of a null deprescribing trial
Source: Implement Sci Commun. 2026 Jun 6;7:148. doi: 10.1186/s43058-026-00983-2 (PMC13464996; doi:10.1186/s43058-026-00983-2)
Supplement: Supplementary file 1 [file 43058_2026_983_MOESM1_ESM.docx]

GRAMMS Reporting Checklist

| Reporting Item | Where in Manuscript |
| --- | --- |
| 1. Describe the justification for using a mixed methods approach to the research question | Methods: A mixed-methods approach was necessary to capture both the qualitative insights from provider comments regarding disagreements with the CDS nudge intervention and the quantitative insights into the associations between the CDS and sentiments. This approach enables us to understand not only the effectiveness of the alerts but also the contextual factors that influence provider decisions and barriers to implementation.” |
| 2. Describe the design in terms of the purpose, priority and sequence of methods | Methods: Comments were first coded (the primary goal) then the quantitative analyses were done to gain insights into the comments (as written in the order of the manuscript). See the subsection “Statistical Analyses.” |
| 3. Describe each method in terms of sampling, data collection and analysis | Methods: Comments were collected naturally in the data (simultaneous sampling). The data pull included both the firing of alerts and the comments. Citation to Braun paper included. |
| 4. Describe where integration has occurred, how it has occurred and who has participated in it | RVNV and HMB independently coded physician comments and then convened to compare their coding. This led to the collaborative determination of primary themes and associated sub-themes. Comments could have been categorized with more than one main theme. The coded themes were classified as either positive (when providers agreed and acted in accordance with the OPA) or negative (when providers did not agree with the OPA).  Citation to Braun paper included. |
| 5. Describe any limitation of one method associated with the present of the other method | Discussion: We address the limitation of the qualitative method (using the natural occurrence of comments – the sampling which can be self-selecting - for quantitative analyses). The analyses have limitations. First, the OPAs' acknowledgment rate was low. Second, given that very few providers left comments within the OPAs, statistical significance can be attributed to noise rather than being representative of the provider population. Further analyses of misplaced or underutilized CDS in an EHR could explore multiple contexts, incorporating variables such as inpatient versus ambulatory care and other disease types to develop a unified tool that evaluates problematic CDS. |
| 6. Describe any insights gained from mixing or integrating methods | The ability to collect contextual insights at the point of care represents a novel and underused method for evaluating and improving CDS systems within a learning health system.    This study demonstrates that EHR comments on CDS can provide valuable evidence regarding how nudges may not affect meaningful clinical behavior changes. We identified key themes surrounding provider resistance to reminders and assessed whether the lack of responsiveness was related to encounter, CDS, patient, or provider characteristics. These contextual variables inform better tailoring of CDS. We contribute to the implementation science literature by offering a method for CDS evaluation to enhance its implementation for healthcare delivery. |

Reference: O'Cathain A, Murphy E, Nicholl J. The quality of mixed methods studies in health services research. J Health Serv Res Policy. 2008;13: 92-98.
